# In memoriam of Prof. Giancarlo Comi

**DOI:** 10.1007/s10072-025-08013-0

**Published:** 2025-02-05

**Authors:** Letizia Leocani

**Affiliations:** 1 Faculty of Medicine, University Vita-Salute San Raffaele, Milan, Italy; 2 Experimental Neurophysiology Unit, INSPE-Institute of Experimental Neurology, IRCCS San Raffaele Scientific Institute, Milan, Italy; 3 Department of Neurorehabilitation Sciences, Casa di Cura Igea, Milan, Italy


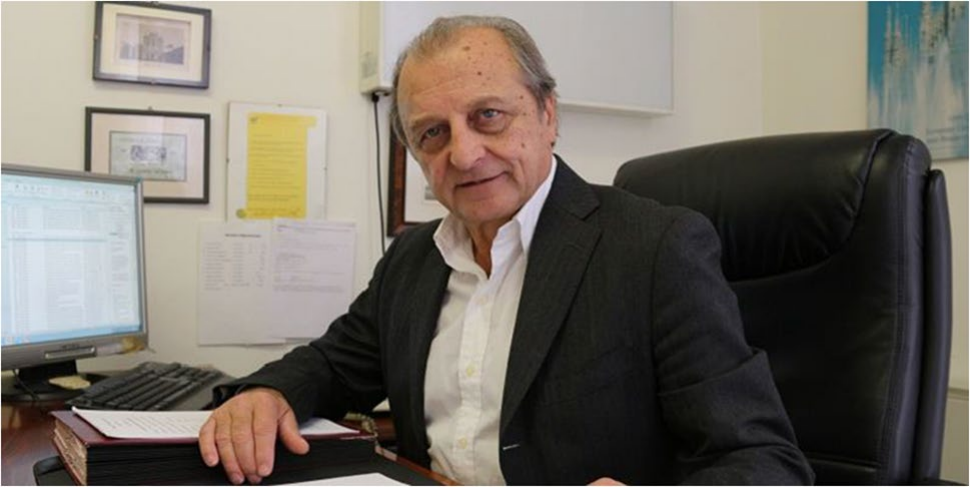


It is with with profound sadness that we learned about the loss of Professor Giancarlo Comi, [Associate Editor of Neurological Sciences since 2010], an extraordinary leader in the neurological community, who passed away on 26 November 2024, at the age of 76.

He was currently President of the European Charcot Foundation, Chair of the *Human Brains* scientific committee of Fondazione Prada, honorary Professor of Neurology at Università Vita Salute San Raffaele in Milan. Moving his first clinical and scientific steps as a clinical neurophysiologist, his work in the field of multiple sclerosis started in 1986, as founder and director of the Multiple Sclerosis Center at San Raffaele hospital.

Author of over 1,000 scientific articles published in the most prestigious international journals and editor of scientific books, he has organized and been a speaker at more than 600 national and international scientific conferences. In 2015, he became the first Italian scientist to receive the Charcot Award for MS Research from the MS International Federation (MSIF).

His numerous recognitions include the *Gheorghe Marinescu* award from the Romanian Society of Neurology, honorary memberships of ECTRIMS, the Russian Neurological Academic Society, the Sociedad Espanola de Neurologia, the Société Francaise de Neurologie, the Gold Medal of *Benemerenza Civica*—*Ambrogino d’Oro*—from the City of Milan and, in 2018, the honour of Officer of the Order to the Merit by the President of the Italian Republic.

As a neurologist and neuroscientist, he combined sharp clinical insight with deep empathy in teaching and patient care. A visionary and passionate physician-scientist, he transformed the therapeutic approach to multiple sclerosis, laying the cornerstone of early treatment of this disease. He started working on multiple sclerosis in 1986, founding the MS Centre at the San Raffaele Hospital in Milan, where he established the Institute of Experimental Neurology in 2004, the first multidisciplinary centre in Italy dedicated to integrating research and treatment of neurological disorders. For this reason, Comi was a leader in promoting the collection of evidence and data that would ultimately lead decision makers to start developing a strategic agenda for the management of this disease. He has been instrumental in countless clinical trials that have contributed significantly to the development of therapies for multiple sclerosis. If we have around 20 drugs today—when only interferons and glatiramer acetate existed in the late 1990s—it is thanks to scientists like him.

Comi was a pioneer in the field of early diagnosis, monitoring and treatment of multiple sclerosis and promoted several paradigm shifts in research and treatment in neurology.

The first was to focus on early pharmacologic treatment of MS, in order to promptly contrast the subtle, silent damage that takes place even before the clinical onset of the disease [1]. Early and effective intervention is now a fundamental milestone in approaching not only multiple sclerosis, but virtually all neurodegenerative diseases.

The second paradigm change was the promotion of early multidisciplinary rehabilitation, based on objective functional markers of damage [2] and outcomes. His focus on the biological effects of rehabilitation in multiple sclerosis has underlined its central role in promoting neuroplasticity [3], improving patients' symptoms and building neural reserve as a preventive strategy for disease progression. With regard to this phase of the disease, Comi was among the promoters of the International Progressive MS Alliance [4], of which he was co-president of the scientific committee in 2015 and subsequently of the Industry Forum.

The third paradigm shift was the concept of *MS care unit*: he was responsible for launching the Multiple Sclerosis Care Unit initiative in 2018 [5], a model for the global management of multiple sclerosis that unites patients, nurses and specialists in a structured and multidisciplinary organisation.

The fourth innovative action in MS care and research was the creation, as president of the International Charcot Foundation, jointly with the International MS Federation and the Italian MS foundation, of the global initiative of Patient Reported Outcome for Multiple Sclerosis [6]. Capturing the experience of people with MS, including their involvement in the creation of their own measures through digital medicine, will make a difference in the management of this disease, leading to preventive and personalized medicine up to brain health.

Following the latter inspiration, Comi also pioneered the brain health vision at the basis of the European campaign *No Health without Brain Health* [7], which aims to prevent neurological diseases, owing to the unique value of the brain capital that must be protected and nurtured. This vision led him to the scientific direction of the Fondazione Prada's *Human Brains* project. It is no coincidence that the new edition of *Preserving the Brain: A Call to Action*, which he promoted as part of the project that Fondazione Prada has dedicated to neuroscience since 2018, focuses on the prevention of neurodegenerative diseases [8].

In each of these roles and initiatives, he has inspired peers and students with his visionary leadership and dedication. His drive to move things forward, his quick thinking, strategic insight and humour made him stand out as a great leader. An indomitable fighter in the face of adversity, he was mentor, guide and role model for countless people, encouraging them with his generous advice and his vision of the future, which he knew how to not only foresee but also to improve.

We will miss him immensely.
